# MicroRNA-199a-5p Affects Porcine Preadipocyte Proliferation and Differentiation

**DOI:** 10.3390/ijms15058526

**Published:** 2014-05-14

**Authors:** Xin-E Shi, Yue-Feng Li, Long Jia, Hong-Lei Ji, Zi-Yi Song, Jia Cheng, Guo-Fang Wu, Cheng-Chuang Song, Qiang-Ling Zhang, Jia-Yu Zhu, Gong-She Yang

**Affiliations:** 1 Laboratory of Animal Fat Deposition and Muscle Development, College of Animal Science and Technology, Northwest A&F University, Yangling 712100, China; E-Mails: xineshi@nwsuaf.edu.cn (X.-ES.); fengfeng0419@163.com (Y.-F.L.); xinongjialong@163.com (L.J.); jihonglei2007@nwsuaf.edu.cn (H.-L.J.); songziyi89@gmail.com (Z.-Y.S.); letitbe521@163.com (G.-F.W.); chengchuangsong@163.com (C.-C.S.); langyan.95@163.com (Q.-L.Z.); jiayuzhulam@163.com (J.-Y.Z.); 2 Vitamin D Research Institute, Shaanxi University of Technology, Hanzhong 723000, China; E-Mail: bobos410@126.com

**Keywords:** porcine preadipocyte, miR-199a-5p, proliferation, differentiation

## Abstract

MicroRNAs (miRNAs), a class of small non-coding RNAs, have emerged as novel and potent regulators of adipogenesis. However, few miRNAs have been fully investigated in porcine adipogenesis, given the fact that pig is not only an apropos model of human obesity research, but also a staple meat source of human diet. In this study, we showed that miRNA-199a-5p is highly expressed in porcine subcutaneous fat deposits compared to several other tissue types and organs measured alongside. Overexpression of miR-199a-5p in porcine preadipocytes significantly promoted cell proliferation while attenuating the lipid deposition in porcine adipocytes. By target gene prediction and experimental validation, we demonstrated that caveolin-1 (Cav-1) may be a bona fide target of miR-199a-5p in porcine adipocytes, accounting for some of miR-199a-5p’s functions. Taken together, our data established a role of miR-199a-5p in porcine preadipocyte proliferation and differentiation, which is at least partially played by downregulating Cav-1.

## 1. Introduction

The entire adipogenic process is initiated by the recruitment of preadipocytes from mesenchymal precursors (adipocyte lineage commitment); under appropriate stimuli, preadipocytes lose the fibroblast-like morphology and differentiate into rounded mature adipocytes (terminal differentiation) [1]. Recent evidence indicated that miRNAs are a class of novel regulators of adipogenesis and actively involved in the two aforementioned phases of adipogenesis [2].

In different stages of adipogenesis, miRNAs can function at the post-transcriptional level by negatively regulating mRNA stability or translation. Liu *et al*. showed that miR-140 is a facilitator of adipocyte lineage commitment [2], whereas several other groups found that miR-27 can suppress the terminal differentiation of preadipocytes by targeting the adipogenic master gene, peroxisome proliferator-activated receptor γ (*PPAR*γ) and prohibitin [3,4]. Other miRNAs, such as miR-17/92 [5], miR-143 [6] and miR-378 [7], are also discovered to play important roles in adipogenesis.

However, since the initial cloning of miR-199a-5p/3p in 2003 [8], the potential roles of miR-199a-5p in adipogenesis have only been superficially investigated. MiR-199a-5p is highly expressed in 3T3-L1 preadipocytes, while its expression declines dramatically once adipogenic induction is present and only rebounds at the late stage of differentiation [9]. Our previous work demonstrated that the subcutaneous adipose tissue (SAT) from piglets has a higher level of miR-199-5p relative to SAT from adult pigs [10]. The expression pattern of miR-199a-5p suggests that it may play a role in adipogenesis, and in fact, studies using human bone marrow mesenchymal stem cells (hMSCs) found that miR-199a-5p overexpression can lead to a ~70% decrease in the mRNA level of *FABP4* (*aP2*), an adipogenic marker [11]. However, to our understanding, there has not been any research dealing with the specific actions and possible targets of miR-199a-5p in fat cells.

In the present study, we established the precise expression pattern of miR-199a-5p during *in vitro* porcine adipogenesis, which is similar to the expression pattern of miR-199a-5p in the 3T3-L1 adipogenic model [9]. Using bioinformatic analyses, we profiled the anti- and pro-adipogenic factors among miR-199a-5p predicted targets, and further experimentation validated that Cav-1 is a bona fide target of miR-199a-5p in porcine adipocytes. We also found that overexpression of miR-199a-5p could attenuate the lipid accumulation during porcine preadipocyte differentiation, which is consistent with the anti-adipogenic effect of Cav-1 inhibition [12]. Furthermore, we used flow cytometry and Hoechst staining to show that the elevated miR-199a-5p level could also lead to accelerated proliferation of porcine preadipocytes. Taken together, our study suggests that miR-199a-5p may be a regulator of porcine adipogenesis, functioning at least partially by targeting Cav-1.

## 2. Results and Discussion

### 2.1. Results

#### 2.1.1. MiR-199a-5p Is Highly Expressed in SAT and Downregulated in Early Porcine Preadipocyte Differentiation

**Figure 1.**
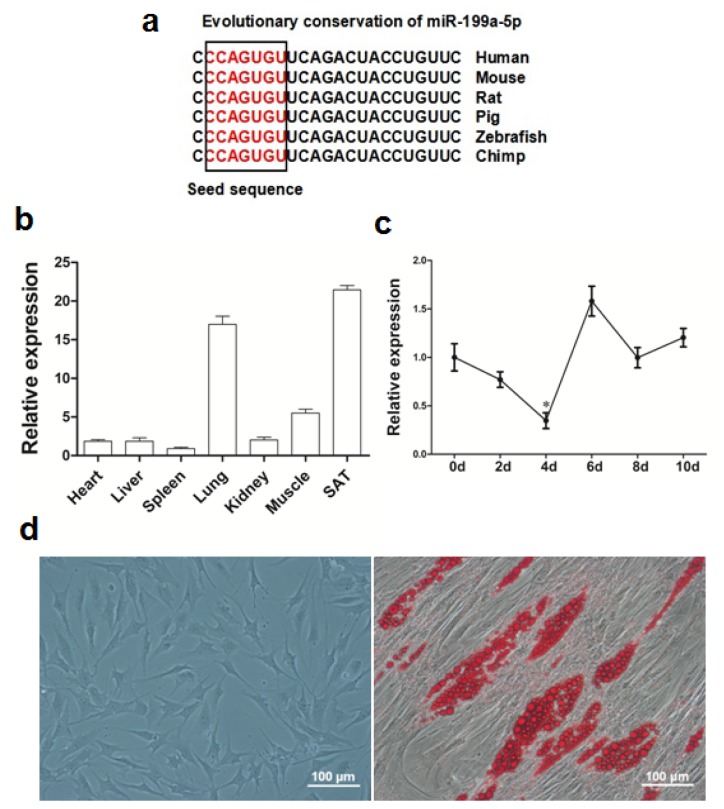
Expression pattern of miR-199a-5p during porcine preadipocyte differentiation and its tissue distribution profile. (**a**) The mature miR-199a-5p sequence is conserved among species; (**b**) porcine tissue distribution of miR-199a-5p; (**c**) the temporal expression pattern of miR-199a-5p during porcine preadipocyte differentiation; (**d**) images of newly isolated and adipogenic differentiated porcine preadipocytes. Quantitative data are represented as the mean ± SEM. *n* = 3. (* *p* < 0.05).

The mature miR-199a-5p sequence possesses high evolutionary conservation among species (Figure 1a). To determine the tissue distribution profile of miR-199a-5p in pigs, the total RNAs were extracted from several types of tissue and organs of adult Guanzhong black pigs. Real-time qPCR data showed that SAT has the highest expression level of miR-199a-5p among the seven tissue types or organs measured (Figure 1b). Previous studies of miR-199a-5p were focused on its function in cardiac and skeletal muscle and several types of oncocytes. However, our results showed that miR-199a-5p is even more abundant in SAT than in heart and skeletal muscle. We proposed that such a high expression level of miR-199a-5p in SAT may have a functional consequence. Based on an *in vitro* porcine adipogenesis model, we observed that miR-199a-5p expression declines by nearly 70% from day 0 to 4 of adipogenic differentiation (Figure 1c). The temporal expression pattern of miR-199a-5p during porcine preadipocyte differentiation is similar to the previous data obtained in the 3T3-L1 model.

#### 2.1.2. MiR-199a-5p Is Capable of Targeting Cav-1 in Porcine Fat Cells

**Figure 2.**
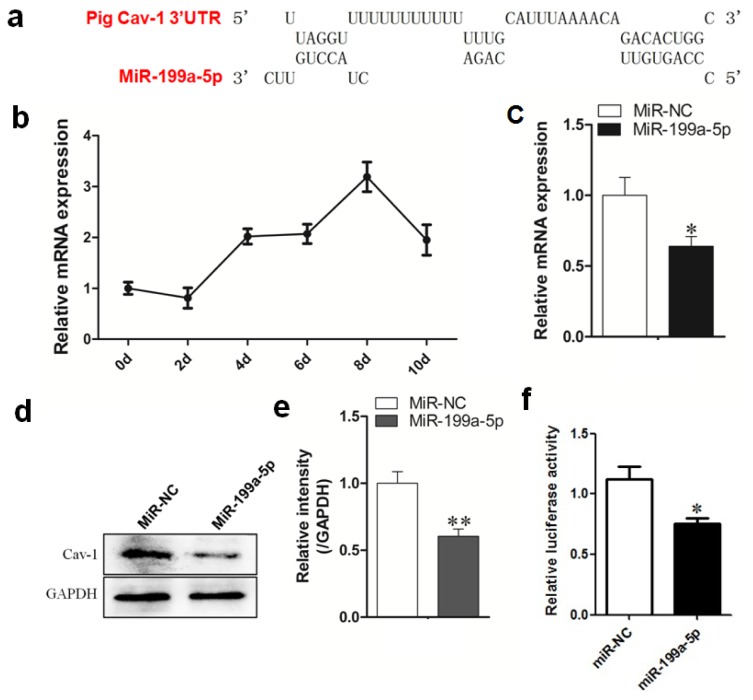
Cav-1 is a direct target of miR-199a-5p in porcine adipocytes. (**a**) A schematic of the targeting site of miR-199a-5p within the Cav-1 mRNA 3′UTR (generated by RNAhybrid) showing that apart from ‘seed sequence’, miR-199a-5p has several other base groups that are also complementary to porcine Cav-1 mRNA 3′UTR; (**b**) the temporal expression pattern of Cav-1 during porcine preadipocyte differentiation; (**c**) the mRNA expression level of Cav-1 in porcine adipocytes upon miR-199a-5p agomir and negative control (agomir NC) transfection; (**d**) protein level of Cav-1 in porcine adipocytes upon miR-199a-5p agomir and agomir-NC transfection; (**e**) is the quantitative representation of (**d**); (**f**) the luciferase reporter assay showing the decreased luciferase activity by miR-199a-5p agomir transfection. Quantitative data are represented as the mean ± SEM. *n* = 3. (* *p* < 0.05, ** *p* < 0.01).

To elucidate the possible function of miR-199a-5p in porcine preadipocyte differentiation, we performed TargetScan 6.2 (http://www.targetscan.org/) and RNAhybrid software to predict the target genes of miR-199a-5p and its interaction schematic with target mRNAs. We also categorized the predicted targets into anti- and pro-adipogenic factors. Among those predicted targets, we noticed that Cav-1 was previously confirmed to be indispensable for fat deposition in mice and pigs. Also, the binding site between miR-199a-5p and Cav-1 mRNA 3′ untranslated region (3′UTR) are conservative among pigs, human, and mice. Both the mRNA and protein levels of Cav-1 were significantly decreased by miR-199a-5p overexpression in porcine adipocytes (Figure 2c–e). We also found that Cav-1 was induced during porcine preadipocyte differentiation (Figure 2b), especially after day 2 of adipogenic induction. Additionally, this expression pattern of Cav-1 is generally inverse to miR-199a-5p, the expression of which was sharply decreased at the early stage of differentiation (day 0 to day 4). We then performed the luciferase reporter assay, and the data showed that miR-199a-5p was capable of binding the 3′UTR of Cav-1 mRNA (Figure 2f).

#### 2.1.3. Overexpression of miR-199a-5p Attenuated the Lipid Accumulation and Adipogenic Marker Genes Expression in Porcine Adipocytes

**Figure 3.**
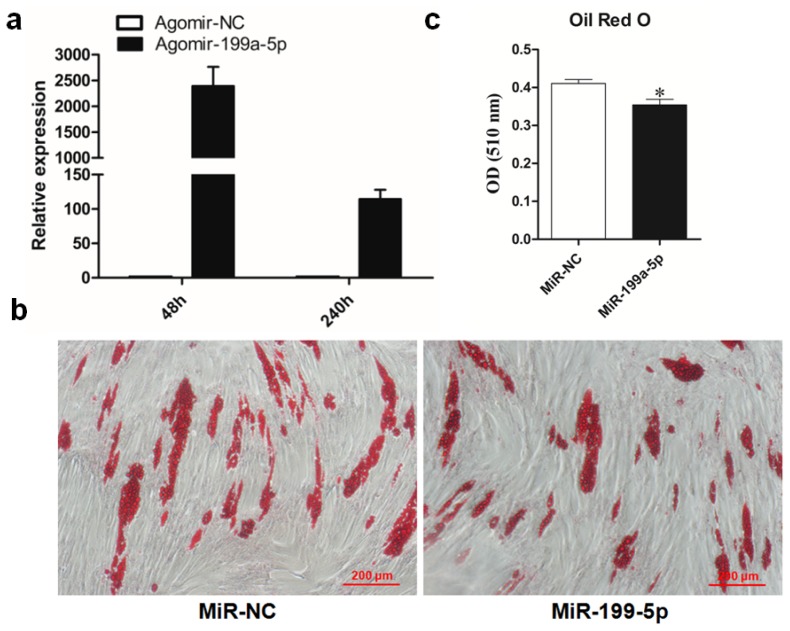
Overexpression of miR-199a-5p impaired lipid accumulation during porcine preadipocyte differentiation. (**a**) MiR-199a-5p agomir or agomir NC were introduced into porcine preadipocytes when cells reached 70% confluency, and 48 h later, cells were subject to adipogenic induction. The overexpression of miR-199a-5p was detected at 48 h through 240 h post-transfection; (**b**) porcine preadipocytes were transfected with miR-199a-5p agomir or agomir-NC and differentiated into mature adipocytes. Cellular lipid droplets were stained with Oil Red O on day 8 of differentiation; (**c**) lipid content indicated as the optical density (OD) value was measured at 510 nm on a spectrophotometer. Quantitative data are represented as the mean ± SEM. *n* = 3. (* *p* < 0.05).

**Figure 4.**
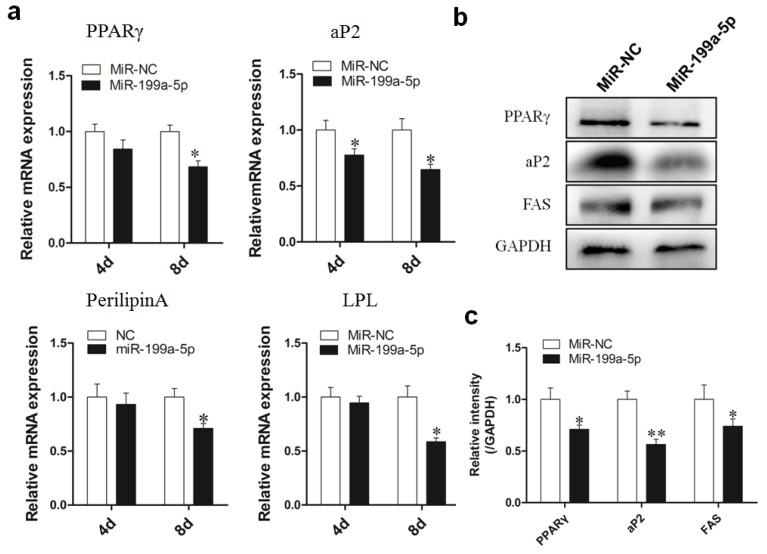
The enhanced miR-199a-5p level in porcine adipocytes downregulated the expression of adipogenic marker genes. (**a**) Porcine preadipocytes were transfected with miR-199a-5p agomir or agomir NC and differentiated into mature adipocytes. Real-time qPCR was performed on days 4 and 8 of adipogenic differentiation to determine the marker genes level; (**b**) Several key markers of adipogenesis were also measured at the protein level using Western Blot on day 8 of differentiation; (**c**) The quantitative representation of (**b**). Quantitative data are represented as the mean ± SEM. *n* = 3. (* *p* < 0.05, ** *p* < 0.01).

Based on the data of the miR-199a-5p expression pattern and target gene validation, we proposed that miR-199a-5p may be a negative regulator of porcine preadipocyte differentiation. To test this hypothesis, we isolated porcine primary preadipocytes from Guanzhong black piglets and performed miR-199a-5p overexpression using synthetic miRNA agomir. The results showed that agomir transfection led to a ~2500-fold overexpression of miR-199a-5p at 48 h post-transfection, and the enhanced miR-199a-5p level could be maintained for at least 8 days after adipogenic induction (240 h post-transfection) (Figure 3a). We then performed the Oil Red O staining and extraction assay and observed that miR-199a-5p overexpression attenuated the lipid accumulation in porcine adipocytes (Figure 3b,c). Real-time qPCR measurement showed that on day 8 of differentiation, the mRNA level of adipogenic marker genes, *PPARγ*, *aP2*, *Perilipin A* and *LPL* were downregulated to different extents by the elevated miR-199a-5p level (Figure 4a). As shown in Figure 4b and c, *PPARγ*, *aP2*, and *FAS* expressions were also tested at the protein level, and Western Blotting of these proteins demonstrated a significant decrease upon miR-199a-5p overexpression.

#### 2.1.4. Overexpression of miR-199a-5p Promoted the Proliferation of Porcine Preadipocytes

**Figure 5.**
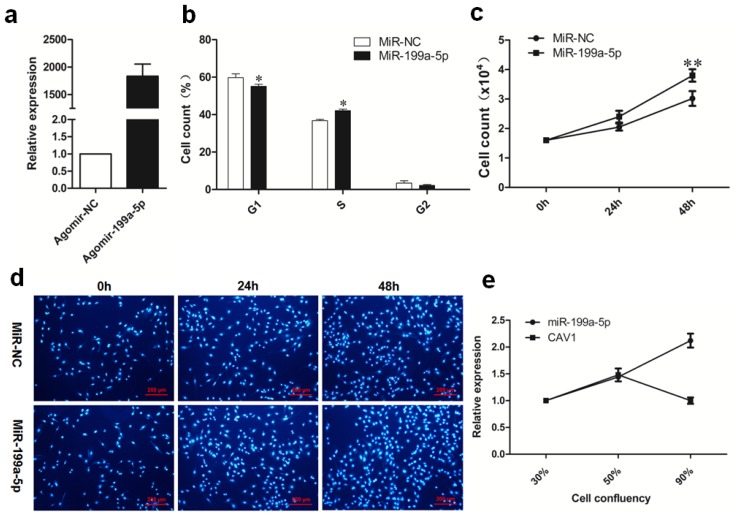
MiR-199a-5p facilitates porcine preadipocyte proliferation. (**a**) Porcine preadipocytes were transfected with miR-199a-5p agomir or agomir NC at 30% confluency. Cells were collected 48 h post-transfection to estimate miR-199a-5p overexpression efficiency; (**b**) porcine preadipocytes were transfected with miR-199a-5p agomir or agomir NC at 30% confluency; 48 h later, the percentage of cells in different cell-cycle phases were determined by flow cytometry. The assay was performed in triplicate and repeated thrice independently, and the original flow cytometry cell cycle charts of one representative experimental repeat were presented in Figure S1; (**c**,**d**) Porcine preadipocytes were seeded in 24-well plates. Cells in each well were counted at 0, 24, and 48 h post-transfection (**c**), and were stained with Hoechst and photographed (**d**); (**e**) porcine preadipocyte cultures were sampled at different cell densities. The expression patterns of miR-199a-5p and its target gene, Cav-1, were measured respectively by real-time qPCR during cell proliferation. Quantitative data are represented as the mean ± SEM. *n* = 3. (* *p* < 0.05, ** *p* < 0.01).

To elucidate whether miR-199a-5p may also function in proliferating preadipocytes, we used flow cytometry to detect the alterations in the cell cycle caused by miR-199a-5p overexpression. The data showed that when miR-199a-5p was overexpressed, the S-phase porcine preadipocytes increased, while the G1-phase cell proportion declined; the change was significant among three independent experiments (Figure 5b). Hoechst staining of the nuclei demonstrated that miR-199a-5p overexpression led to increased cell number 24 and 48 h post-transfection compared to agomir NC treatment (Figure 5c,d). We also tested the miR-199a-5p level at different cell densities after the seeding of preadipocytes. As shown in Figure 5e, the miR-199a-5p expression increased steadily along with the cell number increased, whereas the expression of the miR-199a-5p target, Cav-1, declined as the cell density increased from 50% to 90% (logarithmic growth phase). Therefore, Cav-1 showed an opposite expression pattern to miR-199a-5p during the cell number increased. These results suggested that miR-199a-5p may be a facilitator of porcine preadipocyte proliferation, and its function in proliferative porcine preadipocytes may also be mediated by Cav-1.

### 2.2. Discussion

Pig has been widely accepted to be an apropos research model for human obesity as well as the primary meat stock in many countries. However, studies on miRNAs’ individual function in porcine adipogenesis are quite scarce. In spite of the distinctive expression pattern of miR-199a-5p during preadipocyte differentiation, the possible function of this miRNA in fat development, including porcine adipogenesis, has not been given much attention. In this study, we found that the miR-199a-5p level during porcine preadipocyte differentiation shows a similar fluctuation with that in 3T3-L1 adipogenesis. By the overexpression of miR-199a-5p, we demonstrated for the first time that miR-199a-5p can affect both porcine preadipocyte proliferation and differentiation. Furthermore, we identified Cav-1 as a bona fide target gene of miR-199a-5p in porcine adipocytes.

Apart from Cav-1, many other target genes and sophisticated functions of miR-199a-5p have been intensively studied since miR-199a-5p and -3p were first cloned [8]. In cardiomyocytes, miR-199a-5p is confirmed to directly target hypoxia-inducible factor 1α (HIF-1α) and Sirt1 [13,14], which play important roles upon hypoxia or hypoxia preconditioning of cardiac muscle. In human skeletal muscle, miR-199a-5p is capable of regulating the canonical Wnt pathway and affects myoblast proliferation and differentiation [15]. However, due to the fact that miRNA-mRNA targeting and the interplay relationship differs among tissue and cell types and physiological/pathological conditions [16], we proposed that in adipose tissue, miR-199a-5p may have a target gene subset different from other tissue types.

Caveolaes are known as a category of 50- to 100-nm vesicular invaginations distributed on the cell membrane [17]. Caveolin-1, together with another two members of caveolin (Cav-2 and -3), are the most important components and signal molecules of caveolaes. These organelles act as lipid rafts, which account for lipid transport and storage [18]. Among all mammalian tissues, white fat possesses the highest expression level of Cav-1 and the largest quantity of caveolaes [17]. Recently, an increasing number of studies have indicated that Cav-1 is necessary for normal adipogenesis and lipid metabolism. In 2002, Razani *et al*. found that Cav-1-deficiency in mice can render a lean phenotype and resistance to diet-induced obesity [17]. Cav-1 was also shown to directly participate in lipid droplet forming [19]. Studies by Pilch’s group demonstrated that the adipose tissue from Cav-1-deficient mice is composed of smaller adipocytes compared to that from WT mice [20]. Moreover, Cav-1 expression is positively correlated to pig fat deposition, as a study showed that the Cav-1 expression level is significantly higher in the Yacha pigs (a fatty-type indigenous Chinese breed) back subcutaneous fat compared with that of Berkshire pigs (an intensively selected lean-type breed) [21]. In 2012, Zeng *et al*. proved that Cav-1 knockdown significantly impaired porcine adipogenesis *in vitro* [12], which is consistent with our data obtained by miR-199a-5p overexpression.

In addition, we observed that miR-199a-5p can promote porcine preadipocyte proliferation; this is the first time evidence about miR-199a-5p’s effect on preadipocyte proliferation. However, unlike its role in porcine preadipocyte differentiation, we have not determined whether Cav-1 is also the main target gene of miR-199a-5p during porcine preadipocytes proliferate, as no reports thus far have related Cav-1 to preadipocyte proliferation. However, in other cell types, Cav-1 is capable of regulating the receptors of some proliferation associated growth factors, such as the platelet-derived growth factor receptor [22] and the insulin receptor [23]. Transmembrane located Cav-1 serves as a scaffold for the signaling transduction of many pathways. For instance, activated Cav-1 by Src kinase can bridge epidermal growth factor (EGF) and PI3K/Akt pathways, which play crucial roles in regulating cell proliferation [24]. Moreover, a large body of evidence has indicated caveolin-1 as a potent tumor suppressor, which promotes cell-cycle arrest and senescence [25,26], and a decreased caveolin-1 level was frequently observed in several types of cancer [27]. Plus, Vicente *et al*. found that caveolin-1 can modulate the proliferation and viability of HEK293T, ZR75 and NIH3T3 cells by affecting the cell cycle [27]. However, further and direct evidence is still required to determine the role of Cav-1 in porcine preadipocyte preliferation. Such research will better delineate the mechanisms by which miR-199a-5p affects adipogenesis.

## 3. Experimental Procedure

### 3.1. Cell Culture

Porcine preadipocytes were isolated from the cervical subcutaneous adipose tissue of 5-day-old Guanzhong black piglets under sterile conditions. Newly isolated subcutaneous adipose tissue was rinsed in phosphate-buffered saline (PBS). The tissue was then minced and digested with 1 mg/mL of collagenase type I (Invitrogen, Carlsbad, CA, USA) at 37 °C for ~45 min. 200 μm nylon mesh was used to remove the undigested fractions. The filtrated solution was centrifugated at 1500 r/min for 7 min to collect the preadipocytes. The preadipocytes were then resuspended and washed three times using PBS and, finally, seeded in growth medium (Dulbecco’s modified Eagle’s medium (DMEM; GIBCO, GrandIsland, NY, USA)/F12 (GIBCO) supplemented with 10% fetal bovine serum (FBS; GIBCO)). For adipogenic differentiation, the cells had to reach confluency. The adipogenic inducer cocktail (0.5 mM 3-isobutyl-1-methylxanthine, 1 μM dexamethasone, and 5 μg/mL insulin) was added into growth medium for 2 days, and then, the preadipocytes were subjected to maintenance medium for another 2 days. Maintenance medium was growth medium supplemented with 5 μg/mL insulin. After the 2-day induction and 2-day maintenance, the medium was replaced by growth medium and changed every 2 days. The whole adipogenic process of porcine preadipocytes took 8–10 days.

293T cells were grown in DMEM supplemented with 10% FBS, and the medium was changed every 2 days to maintain freshness.

Operations on live animals in our study were performed complying with the regulation approved by the Standing Committee of Shaanxi People’s Congress, China. Porcine samples were collected with the approval of the ethics committee of Northwest A&F University. Experimental procedures involving pigs were subject to the supervision of the Animal Care Commission of the College of Veterinary Medicine, Northwest A&F University.

### 3.2. MiR-199a-5p Agomir Transfection

MiR-199a-5p agomir and negative control (NC) are double-strand, designed and synthesized by Genepharm (Shanghai, China). The miR-199a-5p agomir sequence is listed as follows: sense: 5′-CCCAGUGUUAGACUACCUGUUC-3′; antisense: 5′-ACAGGUAGUCUGAACACUGGGUU-3′, and the agomir NC sequences are: sense: 5′-UUCUCCGAACGUGUCACGUTT-3′; antisense: 5′-ACGUGACACGUUCGGAGAATT-3′.

For transfection followed by adipogenic differentiation, cell density must reach 70% to ensure that the cells can grow to confluency in two days after transfection. Porcine preadipocytes were subjected to serum starvation for 4 h before transfection. MiR-199a-5p agomir and agomir NC were transfected into cells using Lipofectamine 2000 (Invitrogen, Carlsbad, CA, USA). The overexpression of miR-199a-5p was measured 48 h post-transfection and on day 8 of adipogenic differentiation, respectively.

### 3.3. Real-Time qPCR

Total cellular RNAs were extracted using TRIzol reagent (TaKaRa, Otsu, Japan). Approximately five hundred nanograms of the total RNAs were processed into single-strand cDNA using reverse transcription kits. The real-time quantitative PCR reaction was performed in triplicate using SYBR green kits on a Bio-Rad iQ™5 system (Bio-Rad, Hercules, CA, USA). The sequences of primers used for real-time qPCR were listed in Table S1. The internal reference used for MiRNA expression detection was U6 RNA, and β-actin was used to normalize the mRNA expression level. The relative expression level of each RNA was obtained with the 2^−ΔΔ^*^C^*^t^ method.

### 3.4. Western Blot Detection

Porcine preadipocytes were homogenized by protein lysis buffer (RIPA, Beyotime, Shanghai, China) supplemented by a protease inhibitor (Pierce, Rockford, IL, USA). The lysates were centrifuged to remove cell debris, and the protein concentration was estimated by the Bicinchoninic Acid assay kit (Beyotime, Shanghai, China). Twenty micrograms of protein equivalents were separated on an SDS-polyacrylamide gel and transferred to a PVDF membrane (CST, Boston, MA, USA). After being blocked in 5% skim milk, the membrane was incubated with primary antibodies at 37 °C for 2 h. Then, the PVDF membrane was washed with Tris-buffered saline with Tween (TBST) and hybridized with a second antibody at room temperature for 1 h. After being washed three times with TBST, protein bands were visualized with chemiluminescence reagents (Millipore, Bedford, MA, USA) and quantified using the Image J program (National Institutes of Health, Bethesda, MD, USA). The PPARγ antibody was purchased from Abcam (Cambridge, UK); aP2, fatty acid synthase (FAS) and GAPDH antibodies were from Santa Cruz Biotechnology (Dallas, TX, USA); the Cav-1 antibody was from Boster (Wuhan, China).

### 3.5. Luciferase Reporter Assay

A fragment of the Cav-1 mRNA 3′UTR, which contains the binding site of miR-199a-5p, was cloned from porcine adipocytes cDNAs using primers tagged with XhoI and NotI cutting sites. The primers used are listed in Table S1. The fragment was cloned into psiCHECK™-2 Vector (Promega, Madison, WI, USA) at the 3′-end of the Renilla gene. The luciferase vector was transfected into 293T cells with miR-199a-5p agomir and agomir NC using Lipofectamine 2000 (Invitrogen). Cells were collected 48 h post-transfection. Renilla luciferase activity was measured and normalized to firefly luciferase activity.

### 3.6. Oil Red O Staining and Extraction Assay

Porcine adipocytes were washed with PBS and fixed in 4% paraformaldehyde at 37 °C for 40 min. After being washed three times, the cell cultures were stained with 1% Oil Red O solution at 37 °C for 30 min. Then, the cell cultures were washed with water prior to visualizing by phase-contrast microscopy. To extract and quantify intracellular Oil Red O, the stained lipid droplets were dissolved with pure isopropanol for 10 min at room temperature. The optical density (OD value) was measured at 510 nm on a spectrophotometer (UNICO, Shanghai, China).

### 3.7. Flow Cytometry

Porcine preadipocytes were seeded in 5-cm dishes (1.6 × 10^5^ cells per dish). 24 h later, after seeding, miR-199a-5p agomir and agomir NC were transfected into porcine preadipocytes using Lipofectamine 2000. Due to the low cell density in the proliferation assay, the preadipocytes were not exposed to serum starvation before transfection. Forty-eight hours after agomir transfection, porcine preadipocytes were harvested by trypsin digestion and washed three times in PBS to remove cell debris. The cells were then fixed in cold 70% ethanol for 2 h, treated with 1 mg/mL RNase A for 40 min at 37 °C and then stained with 20 mg/mL propidium iodide (PI). The cells were subjected to cell-cycle analysis using flow cytometry.

### 3.8. Statistical Analysis

Each experimentation was performed three times independently and each time in triplicate. One-way analysis of variance (ANOVA) in SPSS11.5 software (SPSS Inc., Chicago, IL, USA) was used to perform variance analysis and significance test. Quantified data are represented as the mean ± SEM. *p* < 0.05 is indicated as a significant difference.

## 4. Conclusions

In summary, our data indicated miR-199a-5p to be a facilitator of porcine preadipocyte proliferation whilst being a suppressor of adipogenic differentiation. Cav-1, which is preciously established to be indispensable for adipogenesis in several species, including pigs, was experimentally validated herein to be the target of miR-199a-5p in porcine adipocytes. Based on our current observations and preliminary studies on Cav-1’s role in adipogenesis and cell proliferation, we highly proposed that miR-199a-5p may function as a potential controller of cell proliferation and differentiation during porcine adipogenesis, and such a function may at least partially depend on its regulation on Cav-1.

## Supplementary Information
