# Supplementary material for: MicroRNA-199a-5p Affects Porcine Preadipocyte Proliferation and Differentiation
Source: Int J Mol Sci. 2014 May 14;15(5):8526–38. doi: 10.3390/ijms15058526 (PMC4057746; doi:10.3390/ijms15058526)
Supplement: Supplementary file 1 [file ijms-15-08526-s001.pdf]

Supplementary Information

Table S1. The sequence of primers used for real-time qPCR and amplifying the 3'UTR of Cav-1 mRNA.

Gene	Accession number	Sequence (5'-3')	Product size
perilipin A	NM_001038638.1	F: CAGCCAAGGAAGAGTCAGC<br>R: CCTGGAAGGTGTGTTGAGAG	107 bp
aP2	HM453202	F: GAGCACCATAACCTTAGATGGA<br>R: AAATTCTGGTAGCCGTGACA	121 bp
PPARγ	NM_214379	F: AGGACTACCAAAGTGCCATCAAA<br>R: GAGGCTTTATCCCCACAGACAC	142 bp
LPL	NM_214286.1	F: GGAGAGAGGAAGGGAAAACAGAG<br>R: AGACCGACCAATAAACTGCAAAG	150 bp
β-actin	NM_007393	F: GGACTTCGAGCAGGAGATGG<br>R: AGGAAGGAGGGCTGGAAGAG	138 bp
Cav-1	NM_214438.2	F: ATCCCAAGCATCTCAACG<br>R: AAGAGGGCAGACAGCAAA	157 bp
Cav-1 3'UTR	-	F: CCGCTCGAGAGGCAGTAACGAAAGA<br>R: ATTTGCGGCCGCGAAGGGTAAACTTTGG	708 bp

Figure S1. Original data of one independent flow cytometry assay.

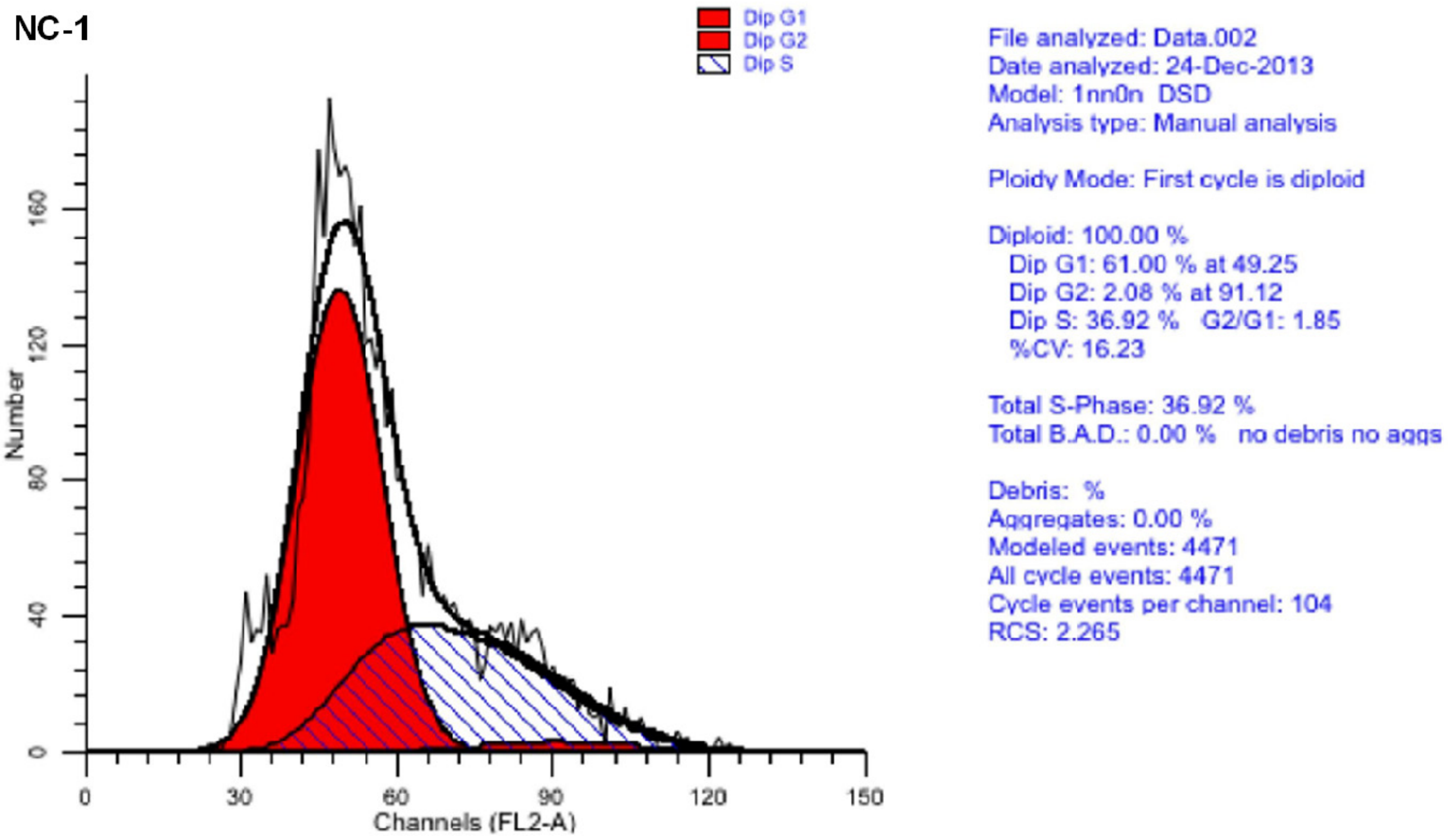

Figure S1. *Cont.*

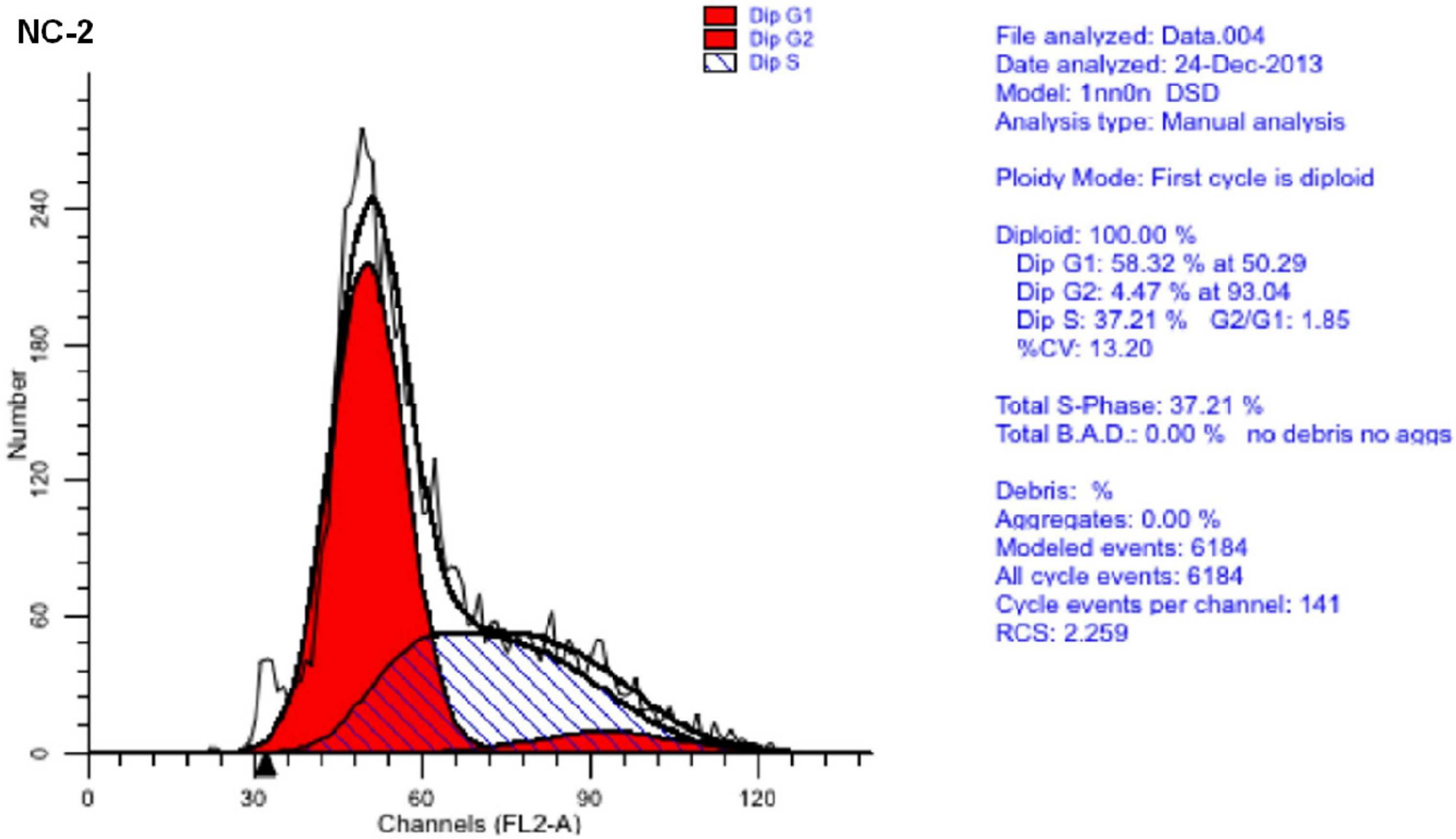

Figure S1. *Cont.*

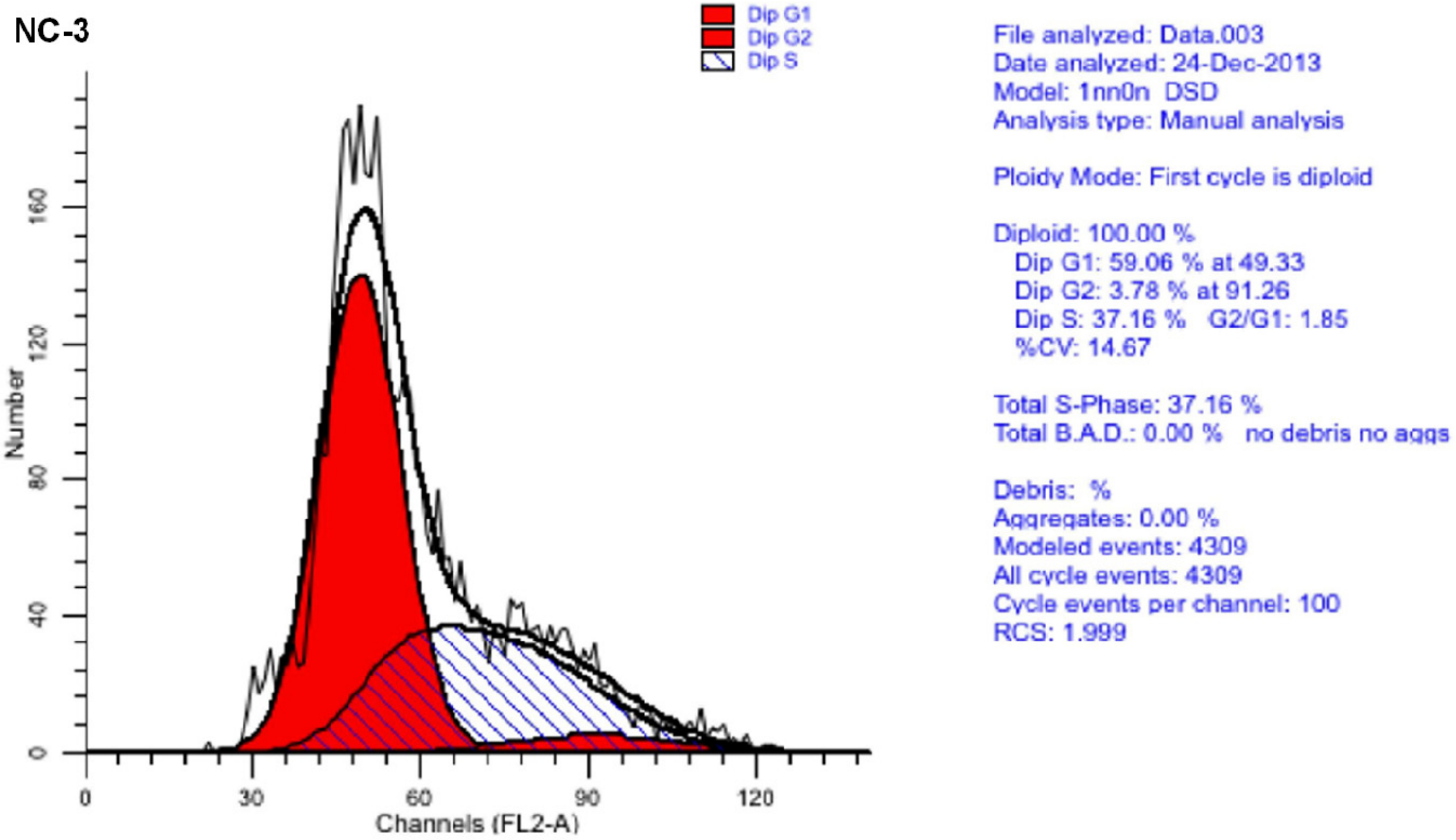

Figure S1. *Cont.*

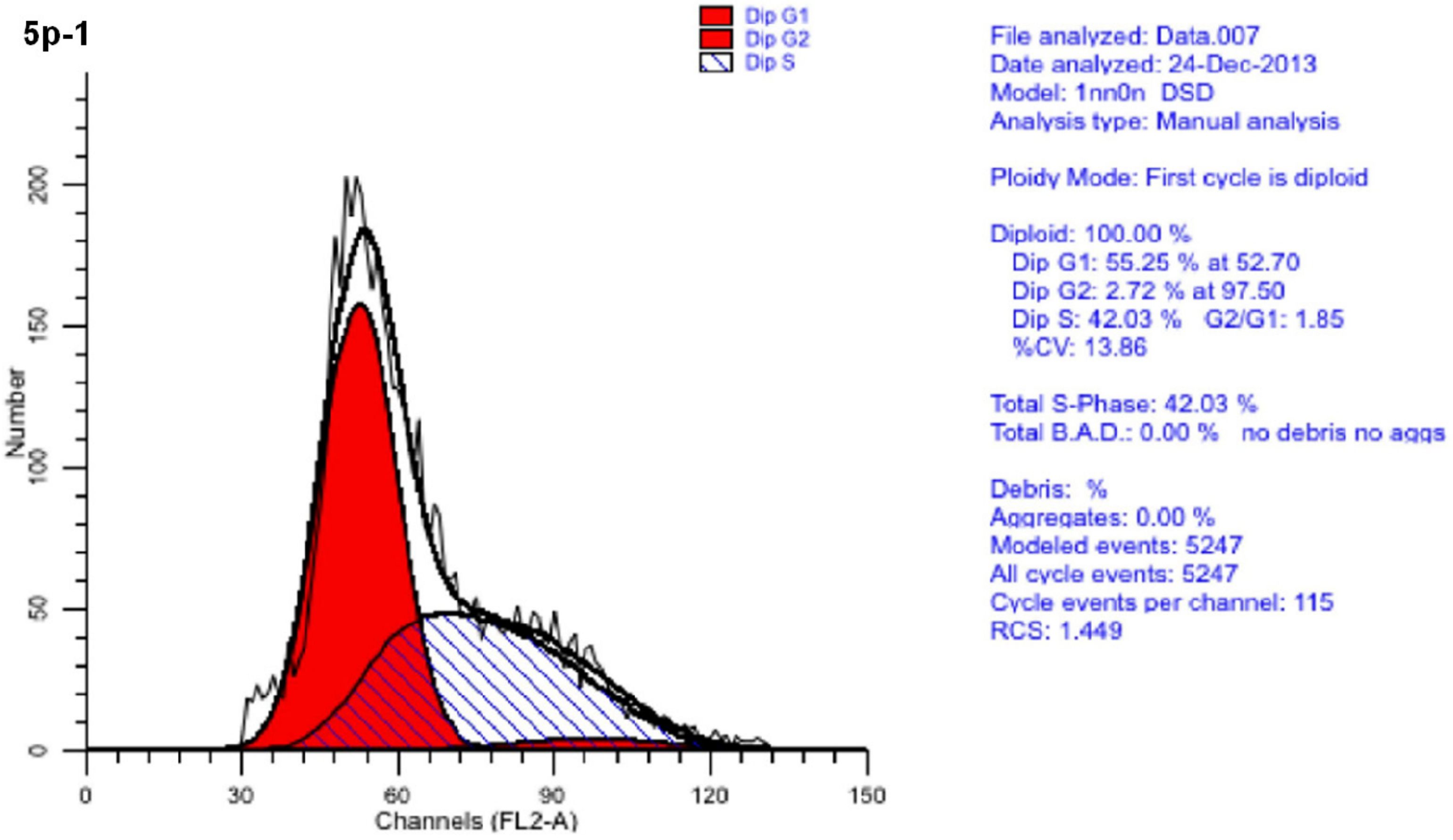

Figure S1. *Cont.*

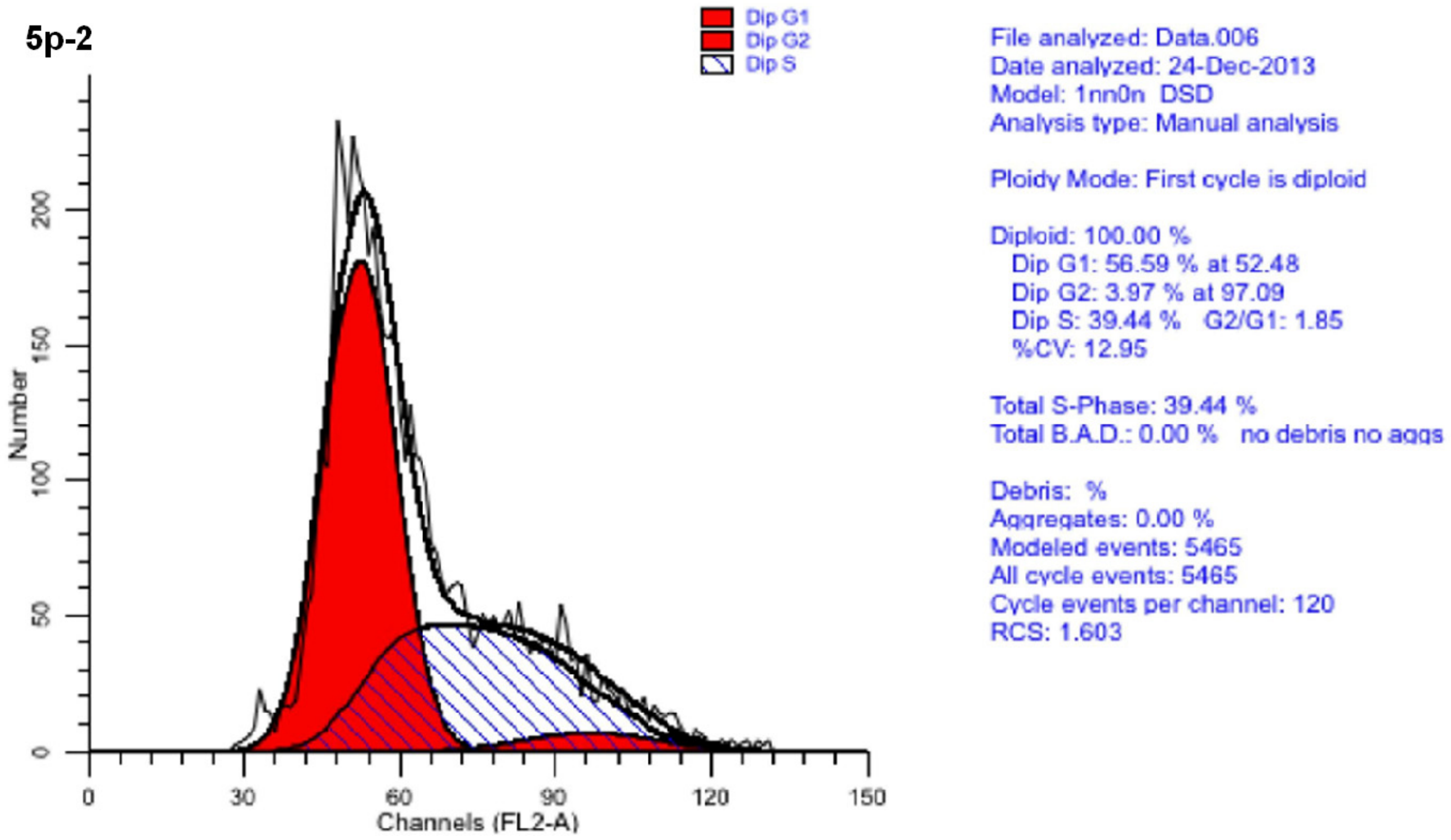

Figure S1. *Cont.*

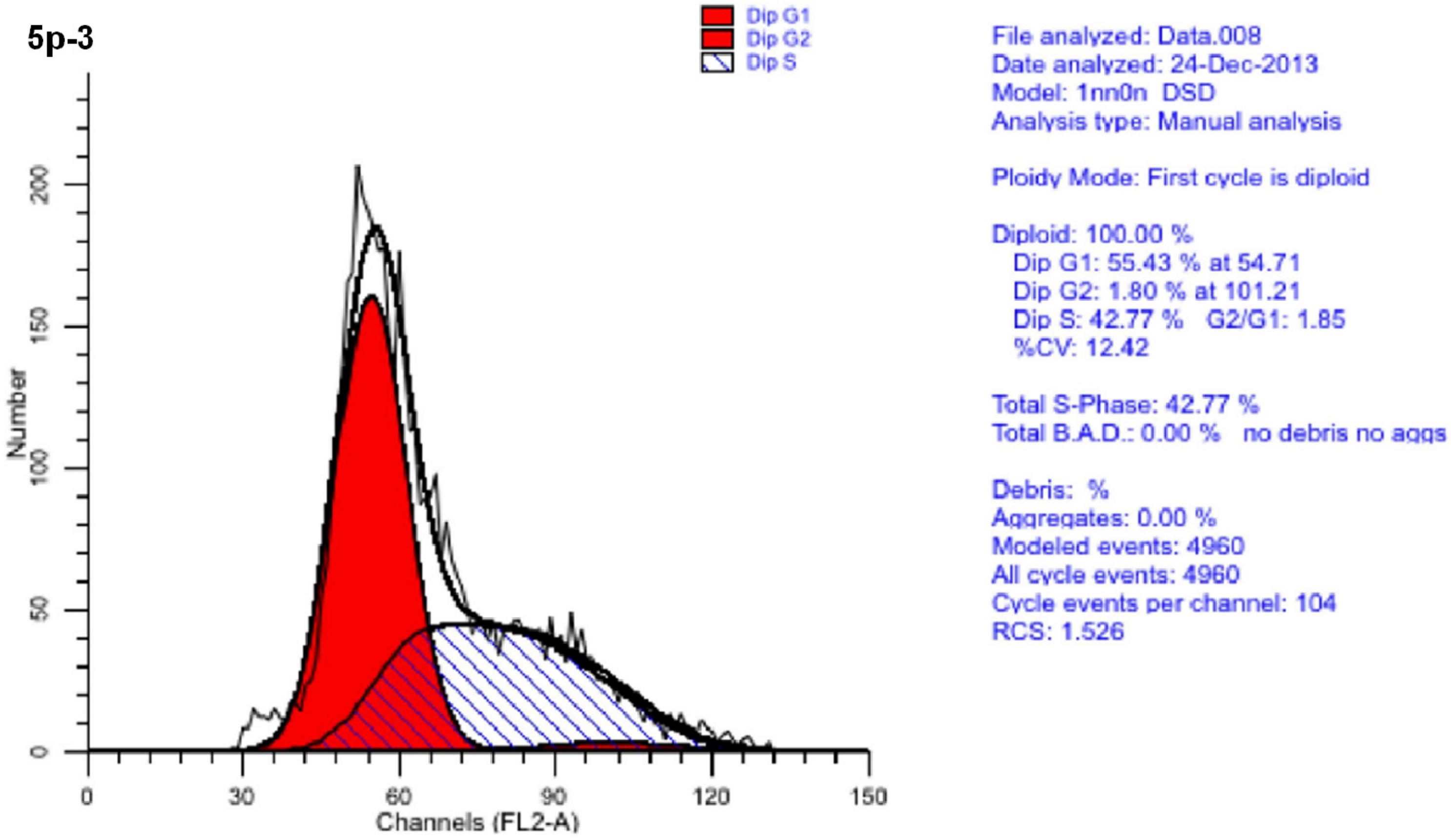

© 2014 by the authors; licensee MDPI, Basel, Switzerland. This article is an open access article distributed under the terms and conditions of the Creative Commons Attribution license (http://creativecommons.org/licenses/by/3.0/).
